# Rash and Eschar in Japanese Spotted Fever

**DOI:** 10.4269/ajtmh.25-0644

**Published:** 2026-03-03

**Authors:** Taiki Nishiba, Kazuhisa Yokota

**Affiliations:** ^1^ Department of Internal Medicine, Mie Prefectural Shima Hospital, Shima City, Mie, Japan;; ^2^ Department of Infectious Diseases, Tokyo Bay Urayasu Ichikawa Medical Center, Urayasu City, Chiba, Japan

## CASE REPORT

**Figure 1.**
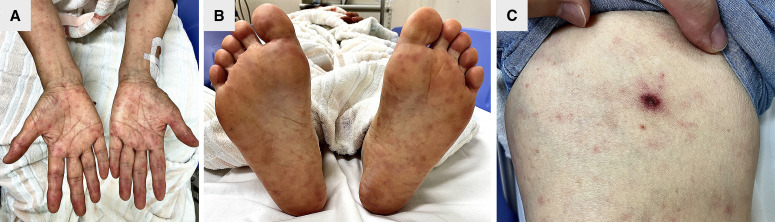
(**A**, **B**) Diffuse erythematous maculopapular rash on the palms and soles. (**C**) A black eschar on the right thigh.

A 76-year-old woman presented with a 3-day history of fever in August. She lived in a rural area of Japan and often worked in her garden. One week before the onset of symptoms, she noticed an asymptomatic black eschar on her right thigh. Upon admission, the patient presented with a diffuse erythematous maculopapular rash involving the trunk, limbs, palms, and soles (Figure 1A and B) and the same black eschar located on the right thigh (Figure 1C). Laboratory tests revealed a white blood cell count of 12.3 × 10^3^/*µ*L, C-reactive protein (CRP) level of 12.1 mg/dL, platelet count of 8.5 × 10^4^/*µ*L, aspartate aminotransferase level of 46 U/L, and alanine aminotransferase level of 40 U/L. Polymerase chain reaction assays of serum and eschar specimens tested positive for *Rickettsia japonica*. She received oral minocycline for 10 days, and her symptoms resolved at follow-up.

*R. japonica* causes Japanese spotted fever (JSF), a tick-borne rickettsiosis occurring mainly in Japan.^1^ Japanese spotted fever is characterized by high fever, erythematous rash, and a tick-bite eschar.^1^ The primary differential diagnoses for tick- and mite-borne infections in Japan include scrub typhus and severe fever with thrombocytopenia syndrome (SFTS). These diseases share similar clinical features, making early differentiation challenging.^2^^,^^3^ Typically, JSF occurs from spring to summer in western and central Japan, whereas scrub typhus occurs from autumn to early winter across a broader region.^4^ Japanese spotted fever is often associated with higher CRP levels than SFTS, which may aid in distinguishing between them.^3^ Both JSF and scrub typhus are typically treated with tetracyclines such as doxycycline or minocycline, whereas SFTS is a viral illness for which antibiotic therapy is ineffective.^2^^,^^3^

In this case, the presence of rash, eschar, thrombocytopenia, and elevated inflammatory markers during summer in central Japan, where JSF is endemic, strongly indicated JSF rather than scrub typhus or SFTS. Prompt recognition of rashes and eschar with timely treatment is crucial for achieving favorable outcomes.^5^
